# Unmodified Clay Nanosheets at the Air–Water Interface

**DOI:** 10.1021/acs.langmuir.0c02670

**Published:** 2020-12-29

**Authors:** Paulo H. Michels-Brito, Antonio Malfatti-Gasperini, Lina Mayr, Ximena Puentes-Martinez, Rômulo P. Tenório, Daniel R. Wagner, Kenneth D. Knudsen, Koiti Araki, Rafael G. Oliveira, Josef Breu, Leide P. Cavalcanti, Jon Otto Fossum

**Affiliations:** † Department of Physics, Norwegian University of Science and Technology, NTNU, 7491 Trondheim, Norway; ‡ Brazilian Synchrotron Light Laboratory, LNLS, Brazilian Center for Research in Energy and Materials, CNPEM, Campinas 13083-970, Brazil; § Bavarian Polymer Institute and Department of Chemistry, University of Bayreuth, 95440 Bayreuth, Germany; ∥ Department of Physics, University of Boyacá, Boyacá150003,Colombia; ⊥ Northeast Regional Center of Nuclear Sciences, Recife 50740-545,Brazil; # Institute for Energy Technology, IFE, Kjeller 2027, Norway; ∇ Department of Fundamental Chemistry, Institute of Chemistry, University of São Paulo, USP, São Paulo 05513-970, Brazil; ○ Centro de Investigaciones en Química Biológica de Córdoba (CIQUIBIC)-Departamento de Química Biológica Dr. Ranwel Caputto, Facultad de Ciencias Químicas, Universidad Nacional de Córdoba, Córdoba X5000HUA, Argentina; ◆ ISIS Neutron Source, STFC, Didcot OX11 0QX, U.K.

## Abstract

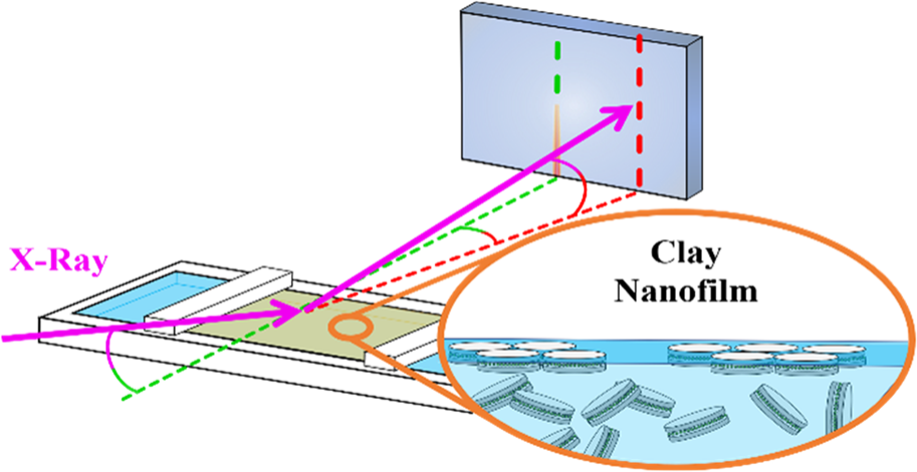

Quasi-two-dimensional (2D) nanolayers, such as graphene oxide or clay layers, adhere to gas–liquid or liquid–liquid interfaces. Particularly, clays are of wide general interest in this context because of their extensive and crucial use as Pickering emulsion stabilizers, as well as for their ability to provide colloidosome capsules. So far, clays could only be localized at oil–water or air–saline-water interfaces in aggregated states, while our results now show that clay nanosheets without any modification can be located at air–deionized-water interfaces. The clay mineral used in the present work is synthetic fluorohectorite with a very high aspect ratio and superior quality in homogeneity and charge distribution compared to other clay minerals. This clay mineral is more suitable for achieving unmodified clay anchoring to fluid interfaces compared to other clay minerals used in previous works. In this context, we studied clay nanosheet organization at the air–water interface by combining different experimental methods: Langmuir–Blodgett trough studies, scanning electron microscopy (SEM) studies of film deposits, grazing-incidence X-ray off-specular scattering (GIXOS), and Brewster angle microscopy (BAM). Clay films formed at the air–water interface could be transferred to solid substrates by the Langmuir–Schaefer method. The BAM results indicate a dynamic equilibrium between clay sheets on the interface and in the subphase. Because of this dynamic equilibrium, the Langmuir monolayer surface pressure does not change significantly when pure clay sheets are spread on the liquid surface. However, also, GIXOS results confirm that there are clay nanosheets at the air–water interface. In addition, we find that clay sheets modified by a branched polymer are much more likely to be confined to the interface.

## Introduction

Self-assembly processes are essential and important in biology, materials science, and technology,^1−5^ for instance, at liquid–liquid interfaces^6−8^ for the design of emulsions. Particle-stabilized Pickering emulsions^9−12^ are widely used in various fields,^10,13,14^ pharmaceutical, agricultural, food, oil recovery, cosmetic, electronic, polymer, processing industries, etc., and has been the focus of intense interest and research. However, open questions related to how complex molecules interact and assemble at interfaces remain to be resolved, and this is in particular the case for assemblies of two-dimensional (2D) layers at fluid–fluid interfaces.^14^

Two-dimensional materials such as graphene oxide (GO) layers in aqueous suspension can self-assemble at different interfaces (air–liquid, liquid–liquid, and liquid–solid).^15^ Various methods have been employed for studies of self-assembly of GO at the air–liquid interface such as Langmuir–Blodgett methods for single-layer film assembly, evaporation-induced assembly for free-standing membranes, or three-dimensional (3D) interfaces for crumpled shells.^15^ At liquid–liquid interfaces, breath-figure^15^ assembly for polymer/GO hybrid honeycomb structures has been studied. Three-dimensional interfaces in Pickering emulsions have been used for production of capsules based on GO, including liquid–solid interfaces, such as on nanoparticle surfaces or assembly at ice–water interfaces.^15^

Smectite clay, graphene oxide (GO), and graphene nanolayers are all examples of 2D materials employed for stabilization of Pickering emulsions^16−25^ or colloidosomes.^26−29^ These nanolayers can be produced from bulk materials that delaminate in layers with about 1 nm thick single layer (SGL) for clay^30^ and about 0.8 nm for single-layer GO.^31^

**Figure 1:**
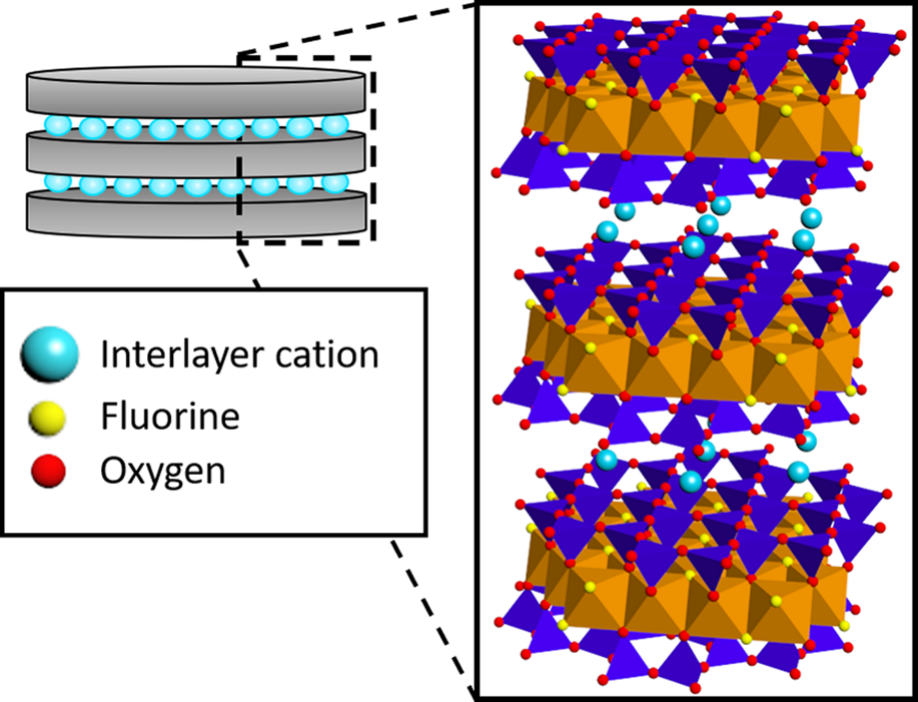
2:1 layered phyllosilicate fluorohectorite clay mineral particle and the corresponding schematic representation. The dark blue tetrahedron site is occupied by silicon, the orange octahedron by magnesium or lithium, and the light blue spheres are the interlayer cations, which in the present case are Na^+^.^48^

Clay minerals are widely used materials in the industry and one of the most abundant natural materials on earth.^32^ Clays,^30,33−36^ that are at the base for several applications, present the ability of swelling.^32−35,37^ Synthetic sodium fluorohectorite (Na-FHt) can be produced with a very high aspect ratio, high homogeneity, and charge distribution with superior quality compared to other clay minerals.^38^ It is a 2:1 layered phyllosilicate clay where the structural unit is formed by two inverted silicate tetrahedral sheets, sharing their apical oxygen with one trioctahedral sheet in between.^32,39^ On the trioctahedral sites, a fraction of Mg^2+^ ions is substituted by Li^+^ to generate a structural negative charge,^38,40^ which is compensated by cations attached to the clay surfaces in the interlayer space prior to delamination,^32^ see Figure 1. Synthetic Na-FHt can delaminate in water without the use of external forces such as mechanical.^30,33−35^ These clay minerals, either as stacked flat particles or as delaminated quasi-2D nanolayers, can self-organize in water, forming nematic suspensions with preferential orientation as observed by birefringence, small-angle X-ray scattering (SAXS), or other methods.^30,34,41−46^ In the present article, we use the following nomenclature in accordance with the established standard in the clay community:^47^ a clay tactoid particle is an unexfoliated nanolayered stack and a clay nanolayer is delaminated from such a stack either as a single layer (SGL) or as a double layer (DBL).^35^ A clay film is a collective organization of SGLs, DBLs, or modified versions of these.

Clay minerals are environmentally friendly and nontoxic, in contrast to GO,^49−54^ bringing sustainable advantages to the use of clay, e.g., in surfactant-free Pickering emulsions. The stabilization of Pickering emulsions using clays depends, e.g., on clay particle concentration and ionic strength of the water suspension.^16−19,55,56^ Clay particles modified with polyethylene imine were used for the stabilization of sunflower oil-based Pickering emulsions,^16,26^ and modification with diethylamine or trimethylamine was used for the case of paraffin in water emulsions.^16,57^ Langmuir–Blodgett-based techniques have been used to assemble hybrid clay films^58^ at air–liquid interfaces:^59−62^ Kotov et al. reported the fabrication of ultrathin films of alkylammonium hectorite,^63^ Umemura et al. reported the assembly of ruthenium(II) complex hectorite film at the air–liquid interface,^64^ Hussain et al. reported the formation of a laponite-dye hybrid film,^65^ de Barros et al. assembled organophilic montmorillonite at the air–liquid interface and performed film transfer to quartz substrate,^66^ Koo et al. studied the self-assembly and characterization of organo-montmorillonite at the air–liquid interface,^67^ Struth et al. studied the phospholipid monolayer on a gel surface made with montmorillonite,^68^ and recently Wu et al. built a hybrid clay film using Langmuir–Schaefer deposition.^69^

Unmodified exfoliated smectite clays, such as montmorillonite or bentonite, have been demonstrated to remain in the subphase.^70^ Some groups have reported to observe aggregates of smectite clays confined at the air–water interface using saline suspensions.^18,70−73^

In the present work, we have monitored the structural organization of synthetic fluorohectorite clay nanolayer colloids, modified and unmodified, confined at the air–water interface using surface-sensitive techniques such as grazing-incidence X-ray off-specular scattering (GIXOS),^74^ scanning electron microscopy (SEM), and Brewster angle microscopy (BAM).^75^ Both the film thickness and in-plane organization were studied. The conditions for stabilization of clay films at the interface were studied using a Langmuir trough (LT).^67^ Unmodified SGL and DBL clay were studied as well as modified DBL clay (ModClay). Furthermore, the fabrication of films from modified clay nanosheets was studied in situ by combining DBL clay with a zwitterionic phospholipid at the air–liquid interface and a branched polymer in the subphase.

## Experimental Section

### Materials

Na-FHt (Na_0.5_[Mg_2.5_ Li_0.5_]⟨Si_4_⟩O_10_F_2_) was synthesized from the melt following a published procedure,^38^ resulting in clay with high aspect ratio and unique homogeneous charge density. This clay mineral has been demonstrated to have superior quality compared to other clay minerals.^38^

The NH_4_Cl, ReagentPlus, ≥99.5%, purchased from Sigma-Aldrich was used in ordered interstratification to obtain DBL, and branched 20% ethoxylated polyethylenimine (PEIE), with molecular weight 20 000, was purchased from BASF and used to modify DBL clay following the procedure given in ref (35).

The zwitterionic phospholipid dipalmitoyl phosphatidylcholine (DPPC) at ≥99% used as a lipid surfactant was purchased from Sigma-Aldrich for GIXOS and SEM experiments and from Avanti Polar Lipids for BAM experiments and used without further purification. Chloroform, EMSURE ACS, ISO, Reagent Ph Eur, was purchased from Sigma-Aldrich. Si wafers for SEM imaging were purchased from Siegert Wafer. Si wafers’ specifications are as follows: grade, prime; CZ growth; B dopant, orientation, 100; resistivity, 1–5 Ω/m.

### Methods and Sample Preparation

#### Langmuir Monolayers and Film Transfer

A Kibron Langmuir–Blodgett trough model MicroTrough XS with two symmetric barriers and a dip coater Layer X on top of a passive antivibration system was used to make Langmuir films at NTNU. The Langmuir trough was filled with deionized water (Milli-Q), and the surface was thoroughly cleaned and monitored to keep the maximum pressure of the isotherm below 0.5 mN/m. Since the clay was suspended in water, 2.0 mL of suspension was carefully spread on the surface and the amount of material was always above the quantity that segregates from the bulk and stays at the interface. The clay film was controlled by the amount of material on the active surface area between the two barriers. The surface pressure was always zero (the same as of deionized water). Clay modified by PEIE (M) on one surface and DPPC (L) on the opposite surface makes MLClay, where now the two external surfaces of DBL are modified differently. This was done in three steps: First, the DBL suspension was spread on the surface, followed by spreading of the DPPC lipid solution on top of the clay films while continuously monitoring the pressure to keep it around 30 mN/m. Modification of the DBL while residing at the air–water interface restricts the adsorption of DPPC to one external surface of DBL yielding Janus-type colloids (LipClay). Next, 400 μL of PEIE solution was inserted into the subphase by carefully introducing a 100 μL Hamilton syringe needle underneath the liquid surface between barriers, thus producing the MLClay.

The Langmuir films were transferred onto Si wafers by the Langmuir–Schaefer method (LSM)^76−79^ for SEM imaging. LSM was chosen in order to transfer only the Langmuir film, and avoid deposition from the subphase. The wafer surfaces were moved to the film at a constant speed of 1 mm/min, kept at the air–liquid interface for 30 s, removed at a constant speed (5 mm/min), and then dried at room temperature. During the deposition, the interfacial pressure was kept constant at 30 mN/m for samples containing DPPC.

#### Cutting and Cleaning of Substrates

Si wafers were cut to 100 mm^2^ area at the NTNU NanoLab cleanroom ISO-5 using a manual wafer scriber (Süss MA-100). The wafers were cleaned using standard clean-2 (RCA2) method^80^ and immediately before the film transfer we performed a plasma cleaning (Diener Electronics model Femto) using O_2_ or Ar gas in a low-pressure chamber, at 40 W for 10 min at the NTNU NanoLab cleanroom ISO-7.

#### Grazing-Incidence X-ray Off-Specular Scattering (GIXOS)

**Figure 2:**
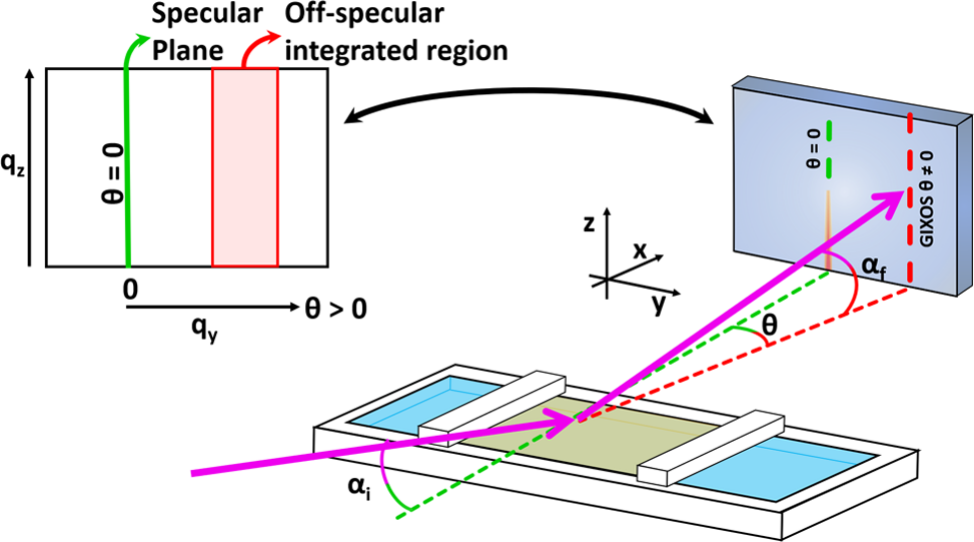
Schematics of the GIXOS technique and active area of the detector.

A NIMA 601 BAM Langmuir trough with two symmetric barriers, on top of an active antivibration system (Accurion), coupled to a surface pressure sensor NIMA-type PS3 with a Wilhelmy plate (paper filter) was used at the Brazilian Synchrotron Light Laboratory (LNLS) synchrotron. In our experiments, the typical Langmuir film pressure ranged from 0 to 40 mN/m. A Langmuir trough, with a maximum area of 600 cm^2^, was mounted on a Huber diffractometer at the XRD2 bending magnet beamline at LNLS. This beamline was equipped for grazing-incidence experiments using a Cr-coated mirror to deflect the beam onto the liquid surface.^81^ The grazing-incidence angle (α_i_) of the incoming beam was set to 0.13°, below the critical angle of 0.15° for the total external reflection from water.^82^ In this configuration, the penetration depth of the X-rays is just a few nanometers below the air–water interface, making this technique very sensitive to surface objects and surface structures. We used Si scatterless slits (Xenocs) before and after the mirror to define the beam, which was set to 150 × 400 μm^2^ (vertical × horizontal). The detector used for GIXOS experiments was a Pilatus 100k (Dectris), and a solid aluminum plate was used as a beam stop, cutting the beam at the position of 0.06° (horizontal θ) from where the off-specular scattering was recorded. The energy was set to 8 keV (λ = 0.155 nm), which gives the maximum flux for this beamline. The sample-to-detector distance was 500 mm. The data is the integration of horizontal angles, a region spanning from θ = 0.06 to 0.14° off specular, depending on the sample, corresponding to 0.04 nm^–1^ < *q_y_* < 0.10 nm^–1^ away from the specular plane, see Figure 2. This is presented as a function of the scattering vector *q_z_* = 2π/λ(sin(α_i_) + sin(α_f_)), where λ is the wavelength, α_i_ is the incident angle, and α_f_ is the scattering angle.

The maximum *q_z_* reached by the setup was close to 6.5 nm^–1^. All experiments were performed at room temperature. The typical total acquisition time per scattering curve was close to 3 h, which was composed of short frames of 1.5 min each. In this way, possible changes during the long scans and changes in sample level due to water evaporation could be monitored. When no change was observed, the frames were integrated to increase statistics. The GIXOS data treatment and off-specular region definition were made using an in-house Python script program and protocols established by the beamline staff.

#### Brewster Angle Microscopy (BAM)

BAM measurements were performed at the University of Córdoba using an EP3 BAM from Nanofilm Technologies mounted on an Accurion antivibration active system. The polarized light in the specular plane comes from a laser of 532 nm (50 mW) at the Brewster angle. A Langmuir mini trough from KVS Instruments was placed with a Wilhelmy plate on top of the microscope for pressure acquisition. The isotherms were measured on deionized water (Milli-Q) and the clay films were controlled by the amount of material and the area between the barriers by ensuring that the surface pressure remained at zero. The DPPC solution was placed on the clay film and the pressure was brought up to 30 mN/m. The images were acquired using a charge-coupled device (CCD) camera with a 10× Nikon lens. The reflectivity was obtained from the gray level of the images after proper calibration.

#### Small Angles X-ray Scattering (SAXS)

SAXS data from DBL suspensions were collected at NTNU using an in-house X-ray scattering instrument equipped with a Pilatus 3 200k (Dectris) detector and a Xenocs X-ray micro-source with a copper anode (energy of 8 keV, λ = 0.154 nm). The sample was measured using a quartz capillary with diameter 1 mm. The scattered X-ray intensities are plotted in terms of *q* (nm^–1^).

#### X-ray Diffraction (XRD)

XRD data of dried films of DBLs were collected at Bayreuth University using PANanalytical X’Pert Pro in Bragg–Brentano geometry with a copper source (energy of 8 keV, λ = 0.154 nm). The sample was prepared by drying a few drops of DBL suspension on a microscope slide at 80 °C for 24 h followed by equilibration in a humidity chamber at 43% relative humidity as set by a saturated potassium carbonate (K_2_CO_3_) solution.

#### Scanning Electron Microscopy (SEM)

SEM data were collected in the cleanroom ISO-5 at NTNU NanoLab using an FEI APREO SEM. SEM measurements were performed at acceleration voltages from 1 to 3 kV, working distances from 4.4 to 10.5 mm, magnifications between 2500 and 15 000 times with a Trinity 2 in-lens detector (T2) for secondary electron signal, an Everhart–Thornley detector (EDT in chamber), and a directional backscattered detector (DBS lens mounted) and biases between 0 and −4000 V. The samples were measured without and with 5 nm coating of a platinum/palladium (80/20) alloy (Cressington model 208 HR B).

#### Sample Preparation

**Figure 3:**
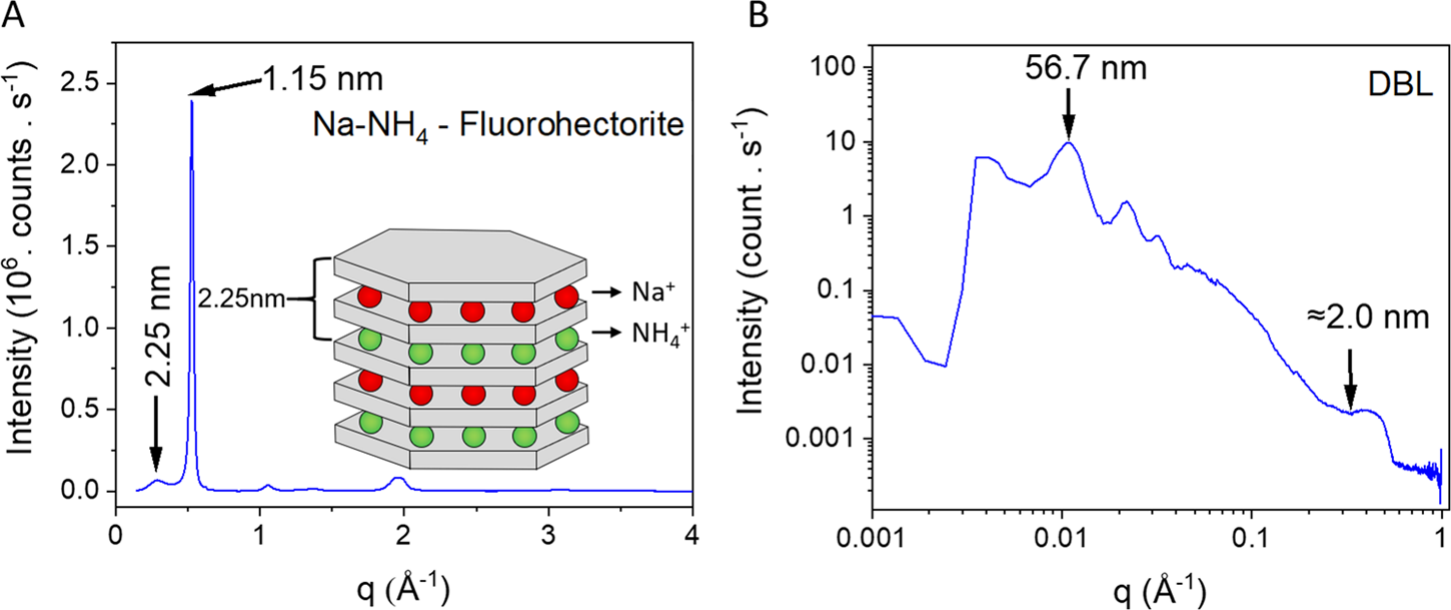
DBL scattering. (A) XRD characterization in Bragg–Brentano geometry of an ordered, crystalline heterostructure clay film equilibrated for 48 h at 43% relative humidity. (B) SAXS of DBLs in water.

Na-FHt delaminates spontaneously upon immersion in water into 1 nm thick single layers (SGLs). To obtain DBLs, first ordered interstratification^35,83^ is produced by partial ion exchange of sodium cations with ammonium cations, resulting in an ordered heterostructure, as confirmed by XRD, Figure 3A. Afterward, the ordered heterostructure was immersed in water, which results in repulsive osmotic swelling of the sodium interlayer yielding DBLs,^35^ as confirmed by SAXS, see Figure 3B.

Modified clay (ModClay) was prepared using PEIE to functionalize DBLs following the procedure given in ref (35).

The DPPC was diluted in chloroform at a concentration of 0.25 mg/mL stock solution. PEIE was dissolved in water at a concentration of 5 mg/mL at pH 5.0. DBL and ModClay were dispersed in water at a concentration of 0.60 mg/mL each, and the SGL was dispersed in water at a concentration of 0.30 mg/mL.

The samples are described in Table 1 showing the schematic/protocol for each sample and the surface pressure after film deposition.

**Table 1:**
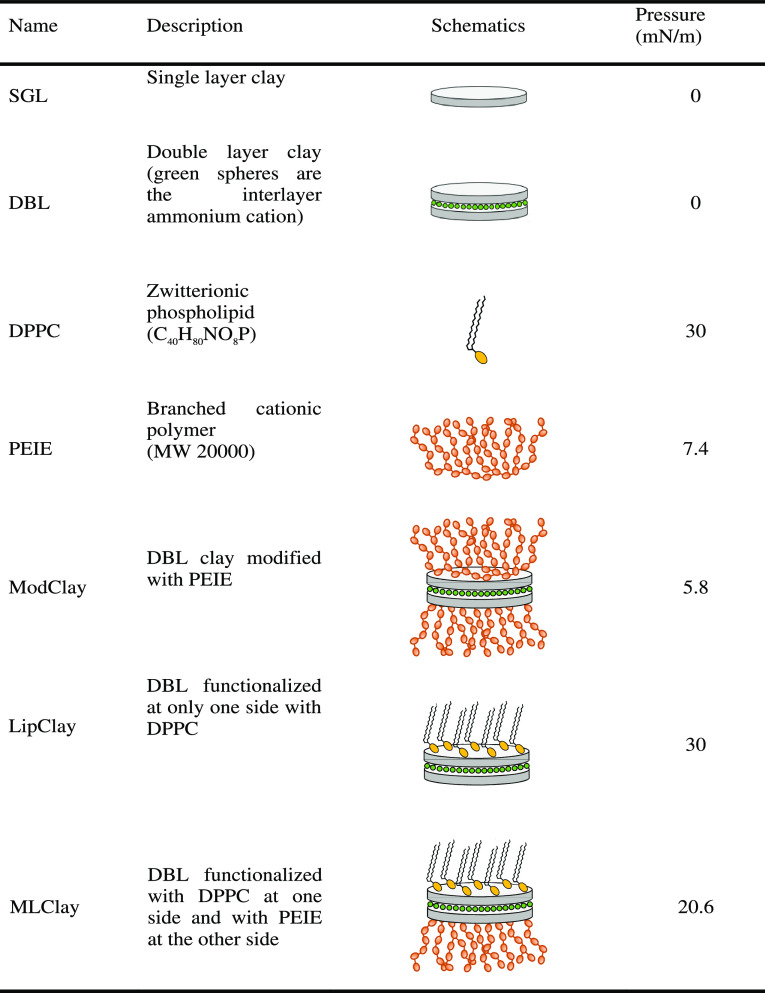
Overview of the Different Samples and the Corresponding Surface Pressure When Spread on Water Surface

## Results and Discussion

We prepared clay thin films at the air–water interface. The interactions of clay, polymer, and lipids were characterized in a Langmuir trough using GIXOS and BAM techniques. Furthermore, the film was transferred from the Langmuir monolayer to a solid substrate to record electron micrographs. The following sections describe each step.

### GIXOS

GIXOS is a surface-sensitive technique that gives information about the electron density (number of electrons per unit volume) profile across the interface.^84^ Here, it was used to get structural information of the organization at the air–water interface. All GIXOS measurements were made using the same trough area (295 cm^2^) without compression of the Langmuir film. The amount of DPPC was adjusted to get a surface pressure of 30 mN/m. For PEIE and ModClay samples, the surface pressure was measured after spreading the samples on the interface.

**Figure 4:**
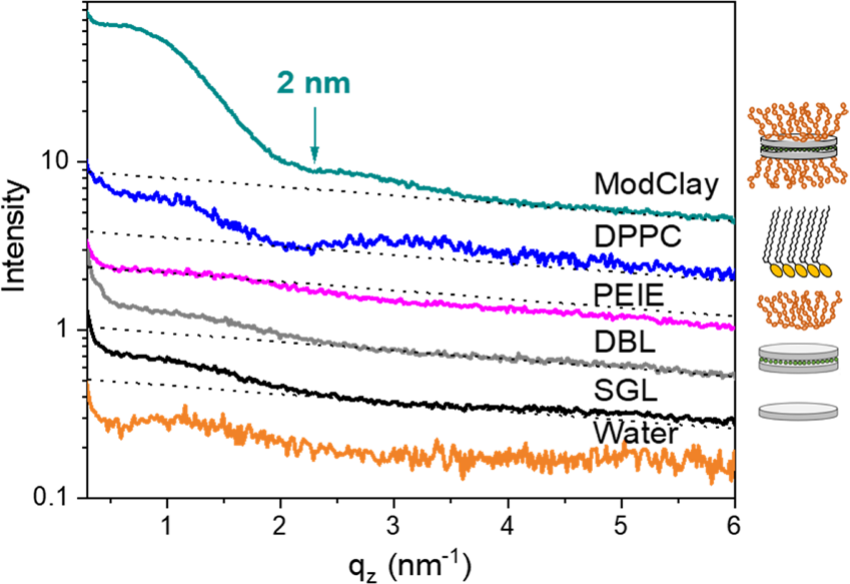
GIXOS intensity vs *q_z_* for ModClay, DPPC, PEIE, SGL, DBL, and deionized water. The black dotted lines represent the behavior of water scattering for comparison. The curves are shifted with respect to one another for the sake of clarity.

**Figure 5:**
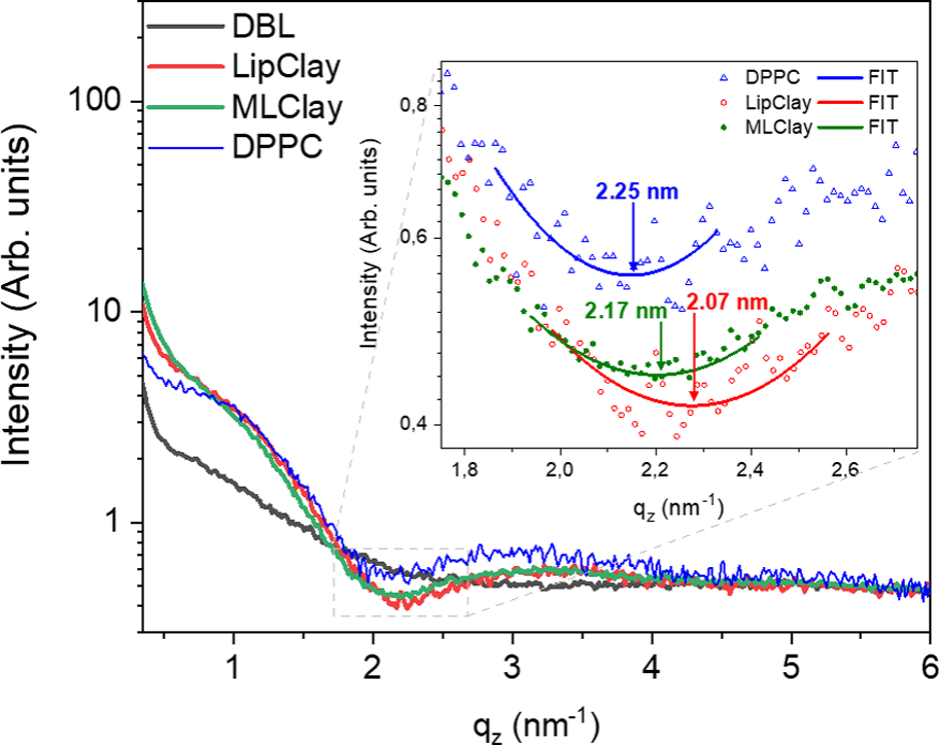
GIXOS intensity vs *q_z_* of DBL, DPPC, LipClay and MLClay nanosheets at constant pressure as in Table 1.

First, we measured GIXOS of deionized water, DBL, and SGL (Figure 4). After spreading the clay samples onto the surface, no surface pressure could be detected using the Langmuir balance. However, GIXOS data suggest that neat clay nanosheets can be confined at the air–water interface even without any modification. Furthermore, the unmodified clays have a small degree of self-organization at the air–liquid interface since the scattered intensity at low vertical angles (*q_z_* < 2 nm^–1^) for both, SGL and DBL, is stronger than for deionized water (which, as expected, presents an almost monotonic value for all *q_z_*’s greater than ≈0.4 nm^–1^, see Figure 4). The difference between deionized water and SGL or DBL scattering is evidence of the presence of unmodified clays at the air–water interface. The increased scattering intensity with respect to the deionized water, however, can only be seen at very low horizontal angles (θ). For horizontal angles higher than 0.17°, the strong scattering at low *q_z_*’s for DBL and SGL fades and these curves show an almost constant value for all *q_z_*’s (greater than critical *q_z_*), similar—but not equal—to the value of water. We believe that this behavior is due to the high aspect ratio of the clay nanosheets (median diameter, 20 μm^38^) in combination with their rigidity, which can affect the evanescent waves present on the water surface, responsible for the off-specular scattering. In comparison, for DPPC monolayers (Figure 4), for example, which is a much softer material, the off-specular signal can easily reach higher horizontal angles (such as 0.4° in our case). Neat DPPC located at the air–water interface (blue curve, Figure 4) at 30 mN/m has a minimum at 2.09 nm^–1^, indicating a thickness—or, more specifically, distance from lipid tails to surface—of 2.25 nm. This is shown in the inset of Figure 5, was obtained from data fitting using a two-slab model for DPPC headgroups and tails. It matches the value obtained from the approximation *d* = 1.5π/*q*_min_,^74,85^ where *q*_min_ is the value where the GIXOS curve has the first minimum. The other values were obtained in a similar way; however, this simple model is not able to fit the increased intensity around 0.5 nm^–1^. The distance is in agreement with previous works^82,86,87^ and is slightly lower than the length (2.8 nm) of DPPC molecules^88,89^ because the chains are tilted.

Neat PEIE also showed a small surface activity. PEIE is a branched polymer and its electron density is very close to the one of water (≈0.345 electrons/Å^3^ for PEIE and ≈0.333 electrons/Å^3^ for water). From the GIXOS signal, the only evidence of PEIE being enriched at the air–water interface is the increase of the scattering decay slope (magenta curve, Figure 4), which is related to the roughness of the surface. However, PEIE presence at the air–water interface is corroborated by the change in surface pressure observed when PEIE was added in the trough. Immediately after spreading PEIE on the air–water interface, the surface pressure reached 7.4 mN/m without applying barrier compression.

It is evident that the ModClay sample (turquoise curve, Figure 4) has a strong scattering at low *q_z_*’s, much more intense than the DBL sample (black curve, Figure 4). This indicates that, as expected, the branched cationic polymer interacts with clay, attaching electrostatically to the clay surface and making it less hydrophilic, thus driving the particle to stay at the air–water interface. The location of the first minimum for the scattering curve of ModClay indicates that the clay segregates from the bulk to the interface and has a thickness of about 2 nm (*q_z_* ≈ 2.34 nm^–1^), which corresponds to the thickness of unmodified DBL. The GIXOS signal is proportional to the square of the difference of the electron densities^84^ and, in the case of the ModClay, comes almost solely from the clay electron density, which is 2 times larger than the one of water or PEIE.

As PEIE is almost invisible by GIXOS due to similar electron density to water, the electron density contrast at the interface is expected to be similar for SGL, DBL, and ModClay (≈0.804 electrons/Å^3^ for the clay) and should be yielding comparable GIXOS scattering curves. The clear difference observed at low *q_z_*’s when comparing SGL and DBL, on the one side, and ModClay, on the other side, is attributed to ModClay staying more firmly at the air–water interface, thus forming a film at the interface due to its increased hydrophobicity upon PEIE modification. This conclusion is corroborated by BAM (see the next section) that showed that SGLs and DBLs fluctuate dynamically between the subphase and the air–water interface. As for DBL and SGL, the GIXOS scattering for ModClay vanishes rapidly as the horizontal angle (θ) increases; for values greater than 0.17° off-specular (*q_y_* = 0.12 nm^–1^), the GIXOS signal is very low, close to the background level, although the intensities for lower angles are high.

The GIXOS experiments were performed in three consecutive steps. First, a DBL dispersion was spread on the air–water interface (black curve in Figure 5). The second step is to spread a DPPC monolayer on the air–water interface and then close the barriers to maintain a 30 mN/m surface pressure (red curve, Figure 5), forming LipClay, see Table 1. Due to the hydrophobic character of the lipid’s tails and the hydrophilicity of the DBL, the DPPC molecules tend to cover the air–water interface pushing back the DBLs.

In the third step, PEIE was added in the subphase. As compared to the GIXOS scattering of LipClay, the changes observed upon attaching PEIE to the second external surface of LipClay (filled green circles, Figure 5) forming the MLClay are small but significant: The position of the minimum on the scattering curve close to *q_z_* = 2 nm^–1^ is slightly shifted (Figure 5, inset) and the profile of the low *q_z_* region (below ca. 0.5 nm^–1^) is altered. Both changes provide evidence for the penetration of the PEIE into the hydrophobic part of the film. Obviously, the adsorption of PEIE on the bottom surface of the DBLs, remote from the interface region, would not be possible to detect in a direct way through GIXOS data analysis since the difference on the electron density of PEIE and water is almost 2 orders of magnitude smaller than that for clay. Furthermore, GIXOS is a surface-sensitive characterization technique, and this layer would be a very small contribution (if any) to the scattering profile.

However, the presence of the increased intensity at low *q_z_*’s shows that (1) the PEIE attachment does not remove the DBLs from the DPPC-DBL layer and (2) the PEIE penetrates the DPPC-DBL layer, changing (slightly) its electron density, which may indicate the tendency of PEIE to interact with LipClay. Functionalization of the bottom DBL surface through the subphase will be discussed below in connection with SEM measurements. We believe that these interactions could be governed by electrostatic attraction between the negatively charged clay surface and the positively charged moieties of the branched polycationic PEIE.

### BAM

The BAM experiments followed the protocols and conditions used in the GIXOS studies, the same stock solutions and dispersions were used producing the same isotherms, and the microscopic patterns from duplicates were highly reproducible. DPPC isotherms were performed, typically showing the following phases: a gaseous (G), a liquid-expanded (LE), and a liquid-condensed (LC) coexistence phase were seen in the form of a plateau region. At a surface pressure of 7.15 mN/m, small patches of phase-condensed regions of DPPC molecules were observed.^87,89,90^ The DPPC islands grew with increasing surface pressure and formed the LC phase when reaching the surface pressure of 15 mN/m.

**Figure 6:**
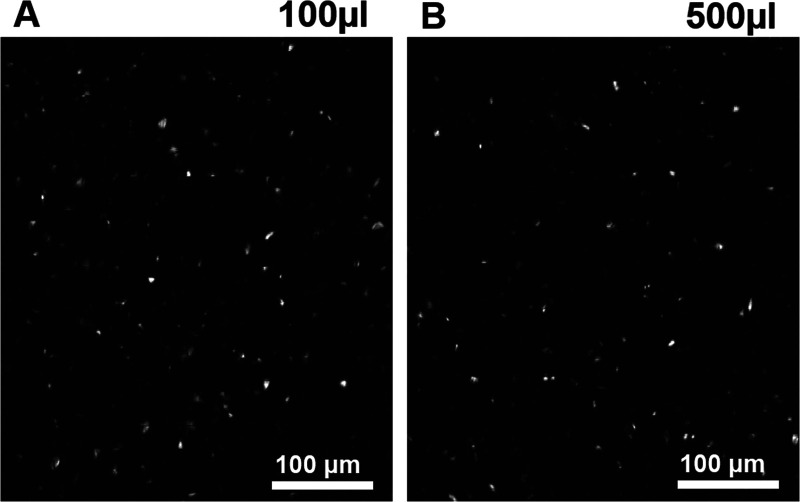
BAM images of DBL clays for different spreading volumes: (A) 100 μL and (B) 500 μL.

In clean deionized water, DBL and SGL stock dispersions were spread drop by drop at the air–water interface in steps of 100 μL aliquots, totaling 500 μL. Regardless of the spread amount of the dispersion, saturated regions as well as layer-populated regions were found, as demonstrated in Figure 6. The SGLs and DBLs move randomly from one place to another, suggesting that clay particles dynamically appear at the interface and return to the subphase, as expected for a dynamic segregation equilibrium, with a tendency of clay to stay longer in the bulk. This dynamic behavior is demonstrated by the movie in the Supporting Information, which shows a marked flickering in reflectivity. The flickering is due to the reaccommodation and loose association of the clay nanosheets at the air–water interface. As already indicated by the ability of crystalline and osmotic swelling, sodium fluorohectorite is very hydrophilic.

**Figure 7:**
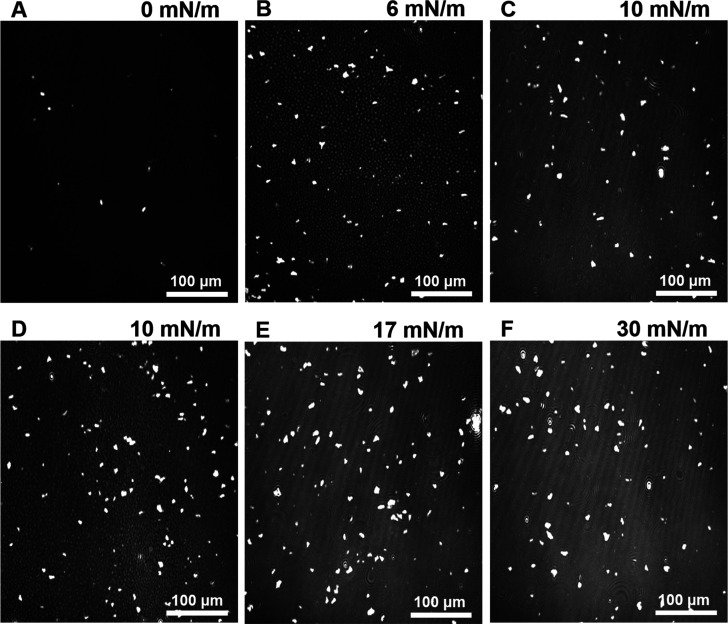
BAM images: addition of DPPC onto DBL in the subphase. (A) G phase at 0 mN/m, (B) continuous LE phase with small LC domains at 6 mN/m, (C) LC phase at 10 mN/m, (D) LE plus LC close to percolation phase at 10 mN/m, (E) LC phase at 17 mN/m, and (F) LC phase at 30 mN/m. Note that at 6–10 mN/m LE and LC phases are coexisting.

Therefore, DPPC was spread over DBL in the trough until a surface pressure of 30 mN/m was reached. During the addition, images were captured at different surface pressures (Figure 7). The DPPC contribution to the images dominates over the DBL, which is attributed to two populations: one from DBLs on the surface, suggesting the formation of LipClay, corroborating with GIXOS results shown in Figure 5, and the other one corresponding to DBLs that reside in the subphase and continue to flicker. At 10 mN/m, LE and LC phases of the DPPC monolayer are coexisting (Figure 7C (LC phase), D (LE phase)). This experimental setup did not allow introduction of PEIE through the subphase. ModClay stock dispersion was spread at the interface in steps of 100 μL aliquots, totaling 500 μL. With each additional step, its concentration increases and the film at the interface becomes more stable.

**Figure 8:**
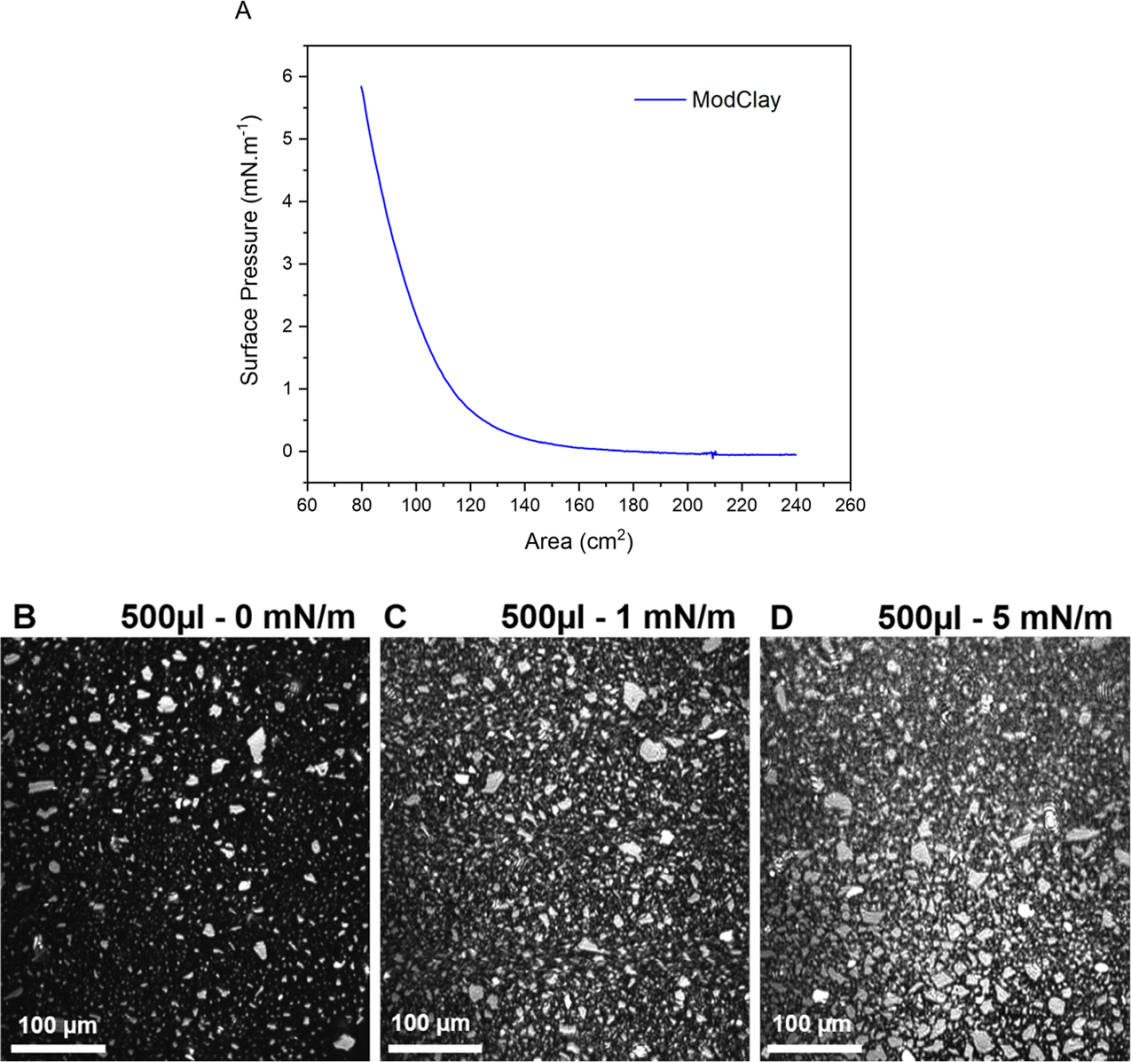
Isotherm and interface snapshots of ModClay in the BAM experiment. The images show ModClay at the interface at different pressures and areas for the same spread volume. (B) 0 mN/m, (C) 1 mN/m, and (D) 5 mN/m.

Figure 8B shows a film with ModClay particle distributed homogeneously after spreading 500 μL of stock dispersion at a pressure of 0 mN/m. Then, the surface area was compressed, reaching a pressure of 5.8 mN/m (Figure 8A), which resulted in a more compact arrangement of the film suggesting some possible collapse (Figure 8C,D shows the interface at different pressures). Like GIXOS, the BAM results demonstrate that ModClay goes to the air–water interface, and this result confirms the GIXOS data of the PEIE curve (Figure 4, magenta curve).

### SEM

**Figure 9:**
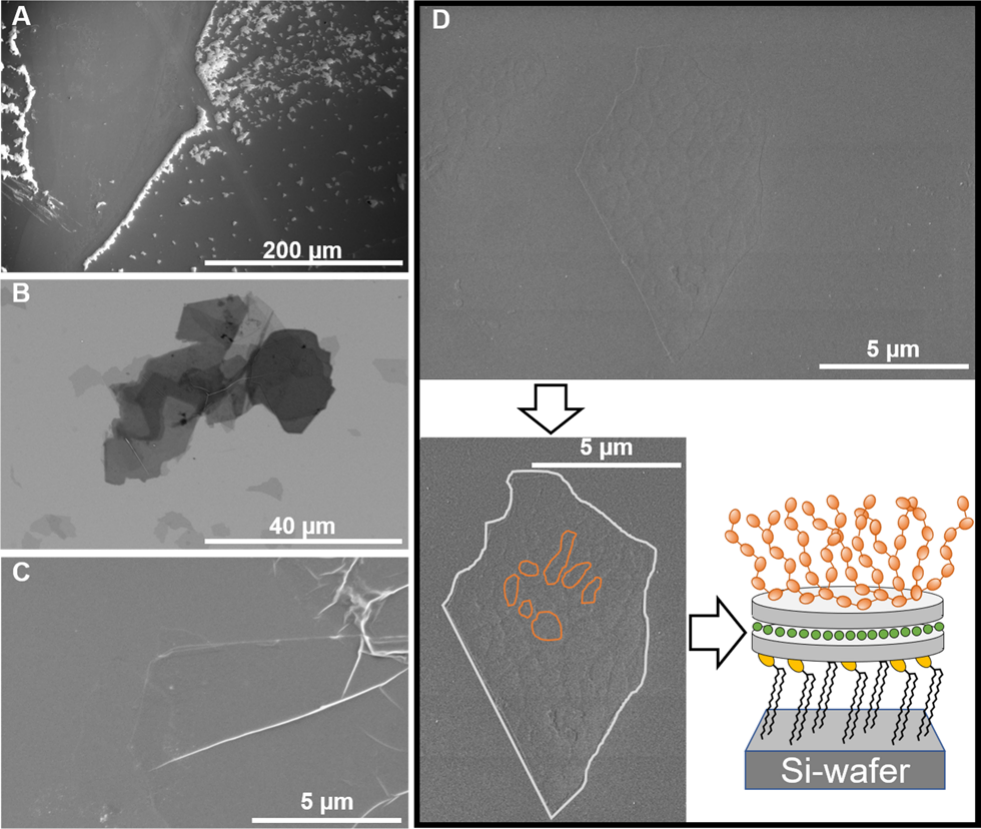
SEM images of each step from DBL via LipClay to possible MLClay. (A) DBL, (B) DBL-stacked clay layers, (C) LipClay, and (D) MLClay, below on the left side is a possible MLClay particle showing the sharp edges and the patchiness of the surface that could be PEIE coiled after drying and on the right side is a schematic of the MLClay on the Si wafer.

Films from each step of the construction process toward MLClay were transferred onto Si wafers by LSM. The transfer was done at least 2 times for each sample to verify the reproducibility that was very good. To observe the film of DBL’s, LipClay and possible MLClay, the samples were sputter-coated prior to the SEM analysis. For DBL, sputter coating was not needed for sufficient contrast. Figure 9 shows the images obtained for DBL, LipClay, and possibly MLClay, where distinct differences were observed for the different samples. For the DBL sample, agglomerates of clay particles were observed, see Figure 9A, and it is explained by the clear tendency of clay to form a “stacked clay layer” arrangement, see Figure 9B, during the drying process. This is because the pure clay nanosheets are not interacting with the substrate via molecules, and the individual clay particles prefer to be surrounded by water that promotes agglomeration of the clay nanosheets. Thus, agglomerates could not be observed in the LipClay film because the clay nanosheets are functionalized by lipid molecules, which then also is evidence for the formation of LipClay, Figure 9C.

Clay particles are well spread on the substrate, which provides a good indication that DBL was functionalized by DPPC, forming the LipClay. Most likely, the DPPC surface of LipClay is the part exposed to the Si wafer, whereas the unmodified surface is free, giving a very smooth, atomically flat surface. In contrast, the MLClay film shows a patchy surface (Figure 9D), which could be caused by adsorption of PEIE on the free side of LipClay. Figure 9D shows the sharp edges of the clay particle and the patchiness of the surface. The drawing suggests how MLClay is assembled on the Si wafer. These results agree with the results suggested by GIXOS.

## Conclusions

This work demonstrates that clay nanosheets made from high-aspect-ratio, superior-quality synthetic fluorohectorite, without any modification or functionalization, can be confined to the air–water interface. BAM results indicate a dynamic equilibrium between unmodified clay nanosheets at the air–water interface and the bulk subphase, probably due to the strong affinity between clay and water. Because of this dynamic equilibrium, the Langmuir monolayer surface pressure does not change significantly when unmodified clay nanosheets are spread on the air–water interface; however, BAM and GIXOS results confirm that the surface has unmodified clay on it. As expected, our results demonstrate that clay nanosheets modified by polymers are even more likely to be confined to the interface than unmodified clay nanosheets. There is a significant difference between the GIXOS scattering intensity from modified clay compared to the scattering from unmodified clay nanosheets on water surface. The thickness of the film on water is determined to be on the order of 2 nm, in the first approximation, agreeing with SAXS/XRD results for DBL particle size. Using the Langmuir–Schaefer method, we succeeded to transfer films from the Langmuir trough to a solid substrate.

The present work is focused on clay nanosheets organization at the air–water interface and is as such foremost relevant for other situations of air–water interfaces such as for clay colloidosome encapsulation of bubbles, or for stabilization of foams. This can also be extended in future work on how clay nanosheets adhere to other types of interfaces such as water–oil or oil–oil interfaces.^91,92^

## Supporting Information Available

Flickering reflectivity of unmodified clay single nanosheets (PDF)
Movie of flickering reflectivity of unmodified clay single nanosheets (MP4)

## Abbreviations

2D: two-dimensional
3D: three-dimensional
SEM: scanning electron microscopy
GIXOS: grazing-incidence X-ray off-specular scattering
BAM: Brewster angle microscopy
GO: graphene oxide
Na-FHt: sodium fluorohectorite
SGL: single layer
DBL: double layer
LT: Langmuir trough
PEIE: branched 20% ethoxylated polyethyleneimine
ModClay: DBL clay modified with PEIE
DPPC: zwitterionic phospholipid dipalmitoyl phosphatidylcholine
LipClay: DBL functionalized at only one side with DPPC
LSM: Langmuir–Schaefer method
RCA2: standard clean-2
LNLS: Brazilian Synchrotron Light Laboratory
T2: Trinity 2 in-lens detector
EDT: Everhart–Thornley detector
DBS: directional backscattered detector
XRD: X-ray diffraction
SAXS: small-angle X-ray scattering
G: gaseous
LE: liquid-expanded
LC: liquid-condensed
