# Supplementary material for: Unmodified Clay Nanosheets at the Air–Water Interface
Source: Langmuir. 2020 Dec 29;37(1):160–70. doi: 10.1021/acs.langmuir.0c02670 (PMC8154875; doi:10.1021/acs.langmuir.0c02670)
Supplement: Supplementary file 1 — la0c02670_si_001.pdf [file la0c02670_si_001.pdf]

Supporting Information

UNMODIFIED CLAY NANOSHEETS AT THE AIR-WATER INTERFACE

Paulo H Michels-Brito[†], *Antonio Malfatti-Gasperini*[‡], *Lina Mayr*[§], *Ximena Puentes-Martinez*[◊], *Rômulo P Tenório*[¤], *Daniel R Wagner*[§], *Kenneth D Knudsen*[†,⊥], *Koiti Araki*[#], *Rafael G Oliveira*[+], *Josef Breu*[§], *Leide P Cavalcanti*[☼], *and Jon Otto Fossum*[†]

† Department of Physics, Norwegian University of Science and Technology -NTNU, Trondheim, Norway
‡ Brazilian Synchrotron Light Laboratory – LNLS, Brazilian Center for Research in Energy and Materials – CNPEM, Campinas, Brazil
§ Bavarian Polymer Institute and Department of Chemistry, University of Bayreuth, Bayreuth, Germany
◊ Department of Physics, University of Boyacá, Boyacá, Colombia
¤ Northeast Regional Center of Nuclear Sciences, Recife, Brazil;
⊥ Institute for Energy Technology – IFE, Kjeller, Norway
Department of Fundamental Chemistry, Institute of Chemistry, University of São Paulo – USP, São Paulo, Brazil
+ Centro de Investigaciones en Química Biológica de Córdoba (CIQUIBIC)-Departamento de Química Biológica Dr. Ranwel Caputto, Facultad de Ciencias Químicas, Universidad Nacional de Córdoba, Córdoba, Argentina
☼ ISIS Neutron Source, STFC, Didcot, UK

The flickering reflectivity is exemplified by this movie of unmodified clay single nanosheets, taken at the same resolution as the static images of BAM (Figure 6), however on a thin strip (close to the red line) where a permanent focus is obtained. Only a horizontal strip is shown in focus because the acquisition times for videos do not allow for the scanning necessary to correct the focus throughout the field of observation, a problem coming from the oblique incidence at the Brewster angle. The field of view in the movie is 190 µm x 190 µm.
